# A 3-D Meandered Probe-Fed Dual-Band Circularly Polarized Dielectric Resonator Antenna

**DOI:** 10.3390/s18082421

**Published:** 2018-07-25

**Authors:** Amir Altaf, Jin-Woo Jung, Youngoo Yang, Kang-Yoon Lee, Sang-Hwa Yi, Keum Cheol Hwang

**Affiliations:** 1 School of Electronic and Electrical Engineering, Sungkyunkwan University, Suwon 440-746, Korea; amiraltafdgu@gmail.com (A.A.); yang09@skku.edu (Y.Y.); klee@skku.edu (K.-Y.L.); 2 Division of Electronics and Electrical Engineering, Dongguk University, Seoul 100-715, Korea; jinwjung@dongguk.edu; 3 Electrical Environment Research Center, Korea Electrotechnology Research Institute, Changwon 51543, Korea; shyi@keri.re.kr

**Keywords:** broadband antenna, dielectric resonator antenna, dual-band circular polarization, hybrid antenna, multi-layer design, 3-D meandered probe feeding

## Abstract

A dual-band circularly polarized (CP) dielectric resonator antenna (DRA) designed on multi-layer substrates is proposed. An asymmetric C-shaped metallic strip is also incorporated into the upper side of the top substrate in the proposed design. The hexagonal dielectric resonator (DR) is excited by the proposed 3-D meandered probe, which generates multiple orthogonal TE-modes. It is found that the lower CP band arises due to the pair of fundamental modes of the hexagonal DR. In the upper CP band, pairs of higher broadside and even modes of the hexagonal DR are combined with a CP band that is induced by the asymmetric C-shaped metallic strip to yield a wide 3 dB axial ratio bandwidth (ARBW). A prototype of the proposed DRA is fabricated for experimental verification. The antenna exhibits a measured −10 dB reflection bandwidth of 56.43% (2.15–3.84 GHz). The far-field measurement shows measured 3 dB ARBWs of 7.56% (2.29–2.47 GHz) with a peak gain of 5.6 dBic and 16.47% (3.12–3.68 GHz) with a peak gain of 7.84 dBic in the lower and upper bands, respectively.

## 1. Introduction

With recent advances in modern communication systems, the demand for an efficient wideband RF front-end is on the rise. Dielectric resonator antennas (DRAs), due to their minimal conduction losses and relatively wide impedance bandwidth characteristics, have captured the attention of antenna engineers as a potential candidate for modern communication systems. Therefore, DRAs with either linearly polarized or circularly polarized (CP) radiation characteristics have been studied extensively for the last three decades. Based on the aforementioned radiation characteristics, CP DRAs provide a flexible orientation between the transmitter and receiver. Moreover, they are resistant to multi-path inteference [1,2,3].

In DRAs, circular polarization is excited by either employing an optimum feeding method or by modifying the shape of the dielectric resonator (DR) such that two orthogonal modes of the same magnitude are simultaneously excited [4,5]. Most previously reported works have concentrated on CP DRAs with a wide 3 dB axial ratio bandwidth (ARBW) [6,7,8,9,10,11]. However, limited data is available with regard to CP DRAs with dual-band circular polarization. Recently, the dual-fed technique was used to excite pairs of fundamental HE${}_{111}$ and higher HE${}_{113}$ modes in a cylindrical DR to yield 3 dB ARBWs of 12.4% and 7.4% in the lower and upper bands, respectively [12]. However, the use of a quadrature coupler increases the complexity, while the matching slots decrease the front-to-back ratio (FBR) as some of the energy escapes through the slots in the backward direction. The single-fed technique, owing to its simplicity, has also been investigated for dual-band CP DRAs [13,14,15,16]. For instance, a singly-fed CP dielectric resonator antenna (DRA) exhibited 3 dB ARBWs of 6.3% in the lower band due to the excitation of a pair of fundamental TE${}_{111}$ modes and 3.68% due to the excitation of a pair of higher broadside TE${}_{113}$ modes in the upper band [13]. Xiao-Chuan at el. introduced a dual-band CP DRA with 3 dB ARBWs of 2.1% and 2.2% [14]. In one study [15], a singly-fed CP DRA with a pair of parasitic arc-shaped slots yielded 3 dB ARBWs of 14.84% in the lower band and 7.11% in the upper band. In another study [16], a dual-band dual-sense CP DRA was designed with 3 dB ARBWs of 15.7% and 6% in the lower and upper bands, respectively. However, all of the aforementioned single-fed CP DRAs [13,14,15,16] have slot-coupled configurations, which decreases the FBR. To solve this problem, a probe-fed CP DRA was presented, in which a wide CP band is split into two CP bands with the help of a metallic strip to yield 3 dB ARBWs of 10.6% in the lower band and 13.5% in the upper band [17]. Nonetheless, no literature has been published thus far on a dual-band CP DRA design which works by the excitation of fundamental and higher order modes along with the absence of backward radiation simultaneously.

In this paper, a dual-band CP DRA excited by a 3-D meandered probe is presented. The proposed feeding method excites three pairs of orthogonal TE-modes in the hexagonal DR separately. An investigation with the dielectric waveguide model (DWM) reveals that the lower CP band arises due to a pair of fundamental TE-modes (TE${}_{111}^{x}$ and TE${}_{111}^{y}$), the central CP band is due to a pair of higher broadside TE-modes (TE${}_{113}^{x}$-like and TE${}_{113}^{y}$-like), while a pair of even TE-modes (TE${}_{121}^{x}$-like and TE${}_{211}^{y}$-like) is responsible for the upper CP band. By placing an asymmetric C-shaped metallic strip at the top of the uppermost substrate, another CP band is induced due to which the pairs of higher broadside and even TE-modes are combined, thus forming a hybrid antenna and yielding a wide 3 dB ARBW at the upper CP band. The ANSYS High-Frequency Structure Simulator (HFSS) was used to perform all of the simulations. The antenna design and the working principle are explained in the Section 2. Section 3 describes the practical evaluation of the proposed design and presents the results of a comparison with earlier works. Section 4 concludes the paper.

## 2. Antenna Design and Analysis

**Figure 1:**
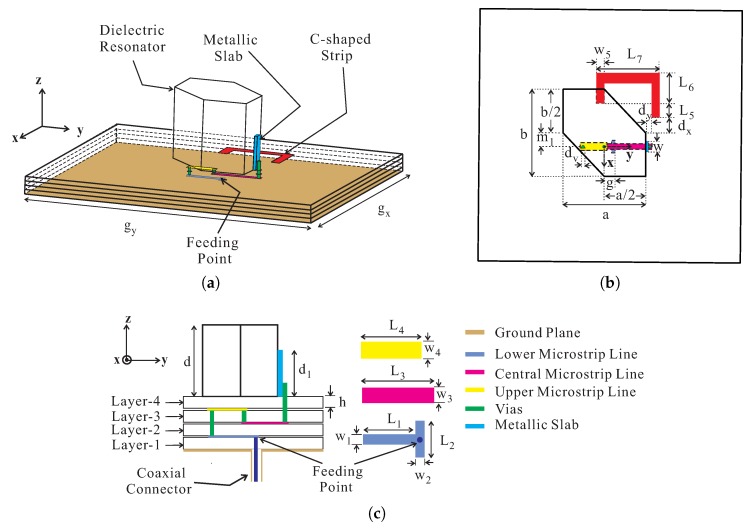
Geometry of the proposed dielectric resonator antenna (DRA): (**a**) 3-D view; (**b**) top view; and (**c**) sectional view.

Figure 1 shows the geometry of the proposed antenna which consists of a hexagonal DR, a ground plane, an asymmetric C-shaped metallic strip, a 3-D meandered probe, and four RF-35 substrates. The four substrates, starting from the lowest to the uppermost, are termed Layer-1, Layer-2, Layer-3, and Layer-4, with each having a relative dielectric constant of 3.5, a loss tangent of 0.0018, and height, *h* [18]. The hexagonal DR with dimensions of *a*×*b*×*d* is made up of alumina (relative dielectric constant $\varepsilon_{d}$ = 10@1 MHz and loss tangent = 0.0002) and is placed at the top of Layer-4. The opposite corners of the hexagonal DR are truncated by b/2 and a/2 along the *x*- and *y*-axes, respectively, while the DR is displaced from the center by distance $m_{1}$ along the -x-axis. An asymmetric C-shaped metallic strip with a width of $w_{5}$ and a *y*-directed length of $L_{7}$ is also placed at the top of Layer-4. The *x*-directed longer leg of a C-shaped metallic strip exceeds the shorter one by a length of $L_{5}$, having a length of $L_{6}$. The -x- and *y*-directed distances from the edge of a DR to the inner edge of the longer leg of the C-shaped metallic strip are $d_{x}$ and $d_{y}$, respectively. The hexagonal DR is excited by the proposed 3-D meandered probe feed that consists of a metallic slab, three microstrip feedlines, and three metallic vias. The lower microstrip feedline has a length and a width of $L_{1}$ and $w_{1}$, respectively, with a tuning stub of length $L_{2}$ and width $w_{2}$ to improve the impedance matching. The distance from the origin to the edge of the stub is *g* with the feeding point at the center. The upper microstrip line of length $L_{4}$ and width $w_{4}$ containing a central microstrip line with dimensions of $L_{3}$×$w_{3}$ lies at the top and bottom sides of Layer-3, respectively. A 1-mm thick metallic slab with width *w* and height $d_{1}$ is utilized to ensure good physical contact and efficient coupling to the hexagonal DR. The microstrip lines and metallic slab are connected through the three vias, each with a diameter of $d_{v}$, while a distance of 0.1 mm is maintained between the edges of the metallic vias and microstrip lines. The ground plane with dimensions of $g_{x}\times g_{y}$ lies at the bottom side of Layer-1. The optimized geometric parameters of the proposed DRA are listed in Table 1.

**Figure 2:**
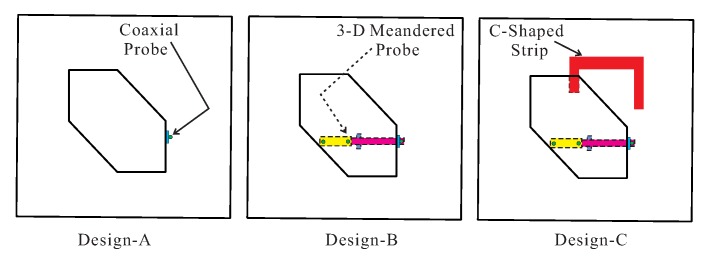
Geometry of Design-A, Design-B, and Design-C.

**Figure 3:**
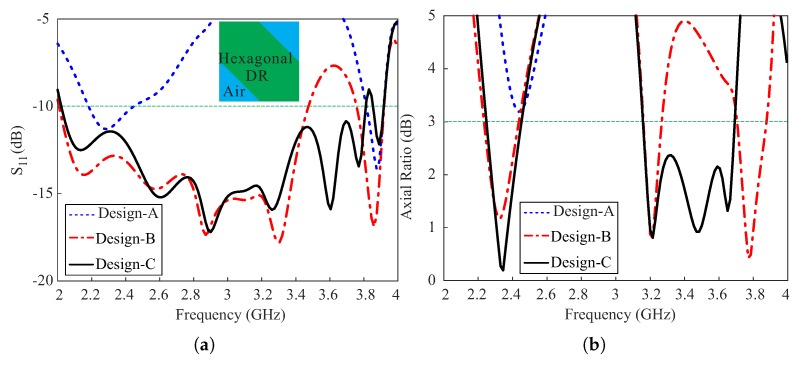
Comparison of Design-A, Design-B, and Design-C in terms of (**a**) the reflection coefficients, and (**b**) the axial ratios.

**Figure 4:**
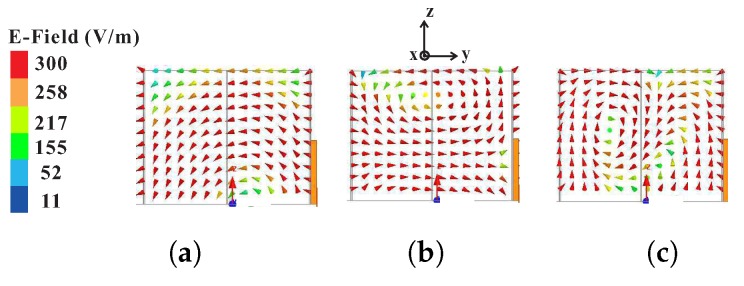
Simulated electric field at the center of a hexagonal DR for Design-B as observed along the *x*-axis at (**a**) 2.32 GHz; (**b**) 3.2 GHz; and (**c**) 3.77 GHz.

To clarify the purpose of the proposed design, three designs—Design-A, Design-B, and Design-C— were compared in terms of −10 dB reflection coefficients and axial ratios (ARs). Figure 2 shows the geometries of these multi-layer, substrate-based designs, and Figure 3 displays the results of the comparison. In Design-A, a conventional coaxial probe is connected directly to the metallic slab, which yields a dual-band response with −10 dB reflection bandwidths of 10.39% (2.19–2.43 GHz) and 1.81% (3.83–3.90 GHz), respectively, as shown in Figure 3a. It should be noted that the coaxial access was configured and taken into account in the simulation of the three designs. As shown in Figure 3b, Design-A exhibits an elliptical polarization around 2.4 GHz as the AR level exceeds 3 dB. In Design-B, the hexagonal DR is excited by the proposed 3-D meandered probe which provides wide impedance matching with −10 dB reflection bandwidths of 52.36% (2.03–3.47 GHz) in the lower band and 3.39% (3.77–3.90 GHz) in the upper band. As indicated in Figure 3b, Design-B exhibits a triple-band CP response with 3 dB ARBWs, lying within the −10 dB reflection bandwidths, of 8.99% (2.23–2.44 GHz), 3.12% (3.16–3.26 GHz), and 2.88% (3.77–3.88 GHz) in the lower, central, and upper bands, respectively. To investigate the reason behind the performance of Design-B, the resonance frequencies of the TE-modes were calculated. Given that the hexagonal DR is obtained by truncating a rectangular DR, the orthogonal TE-modes responsible for this performance are identical to that of the rectangular DR. Therefore, the hexagonal DR was converted into a rectangular DR to find the effective relative permittivity $\varepsilon_{eff}$, as shown in Figure 3a, by applying a simple static capacitance model [19],

(1) $$\varepsilon_{eff}=\frac{\varepsilon_{d}\times V_{dr}+1\times V_{air}}{V_{dr}+V_{air}},$$

where $\varepsilon_{d}$ is the relative dielectric constant of the hexagonal DR and the volumes of DR and air are represented by V${}_{dr}$ and V${}_{air}$, respectively. As the dimensions of the hexagonal DR along the *x*- and *y*-axes differ, the resonance frequencies for a pair of similar modes will also differ. To find the resonance frequencies of TE${}_{1mn}^{x}$-modes, the values of the wavenumber for different modes in the *x*-direction needed to be known. These values were calculated by solving the following transcendental equation for a rectangular DRA placed on a ground plane using the DWM [20]:

(2) $$btan\left(\frac{bk_{x}}{2}\right)=\sqrt{\left(\varepsilon_{r}-1\right)k_{0}^{2}-k_{x}^{2}},$$

where

(3) $$k_{0}^{2}\varepsilon_{r}=k_{x}^{2}+k_{y}^{2}+k_{z}^{2},$$

(4) $$k_{y}=\frac{m\pi }{a},\;m=1,2,\cdots ;\;k_{z}=\frac{n\pi }{2d},\;n=1,3,\cdots ,$$

where $\varepsilon_{r}$ is the relative permittivity. The terms $k_{x}$, $k_{y}$, and $k_{z}$ represent the wavenumbers, while *a*, *b*, and *d* are the dimensions of the hexagonal DR along the *x*-, *y*-, and *z*-axes, respectively. The values of the wavenumber for the TE${}_{m1n}^{y}$-modes can also be calculated in a similar fashion from the above equations. After obtaining the values for $k_{x}$ and $k_{y}$ for the TE${}_{1mn}^{x}$-modes and TE${}_{m1n}^{y}$-modes, respectively, the resonance frequencies of various TE-modes can be calculated with the following equation:

(5) $$f=\frac{c}{2\pi }k_{0},$$

where *c* is the speed of light in a vacuum. The results of the calculated and simulated resonance frequencies for the pairs of fundamental (TE${}_{111}^{x}$ and TE${}_{111}^{y}$), higher broadside (TE${}_{113}^{x}$ and TE${}_{113}^{y}$), and even (TE${}_{121}^{x}$ and TE${}_{211}^{y}$) modes are listed in Table 2. It can be seen that the calculated center frequencies ($f_{c}$) for the pairs of fundamental and even modes suitably approximate the respective simulated values ($f_{simu.}$) given $\varepsilon_{r}$ = $\varepsilon_{eff}$ = 7.75 as calculated from Equation (1). However, there is a considerable difference between the calculated and simulated resonance frequencies of the pair of higher broadside modes. The validity of Equation (1) for a pair of higher broadside modes cannot be determined at the moment, as no conclusive study prior to the proposed work has been conducted. On the other hand, the difference is reduced when using $\varepsilon_{r}$ = $\varepsilon_{d}$ = 10 in Equations (2) and (3). This can be thought of as if the hexagonal DR with a smaller volume is operating at the same frequencies for a pair of higher broadside modes as a rectangular DR, as depicted in Figure 3a, of a larger volume, provided that both instances of $\varepsilon_{r}$ = $\varepsilon_{d}$ are true. Thus, the proposed antenna has achieved a miniaturization for a pair of higher broadside modes. To ensure the generation of the aforesaid modes, the simulated electric fields were observed at the center of the hexagonal DR for the simulated AR minima of Design-B, as depicted in Figure 4. It is evident that the mode distributions at 2.32 GHz, 3.2 GHz, and 3.77 GHz resemble the TE${}_{111}^{x}$, TE${}_{113}^{x}$-like, and TE${}_{121}^{x}$-like modes of a rectangular DR.

For Design-C, by adding an asymmetric C-shaped metallic strip at the top of Layer-4 of Design-B, the widest −10 dB reflection bandwidth of 61.2% (2.03–3.82 GHz) is achieved due to two additional resonance bands around 3.6 GHz and 3.75 GHz, as shown in Figure 3a. As shown in Figure 3b, the purity of circular polarization at the lower CP band improves but the 3 dB ARBW remains nearly identical to that of Design-B. On the other hand, a wide 3 dB ARBW of 15.47 % (3.16–3.69 GHz) is obtained at the upper band from the merging of the pairs of higher broadside and even modes with a CP band induced by the asymmetric C-shaped metallic strip around 3.47 GHz. The asymmetric C-shaped metallic strip was utilized in the proposed design because it readily facilitates the generation of rotating currents. The final dimensions of the C-shaped strip were chosen through a genetic algorithm optimization technique; the mean length of the C-shaped strip was 44.5 mm, which is approximately half of the wavelength at 3.47 GHz. Therefore, the proposed antenna acts as a hybrid antenna at the upper CP band.

**Figure 5:**
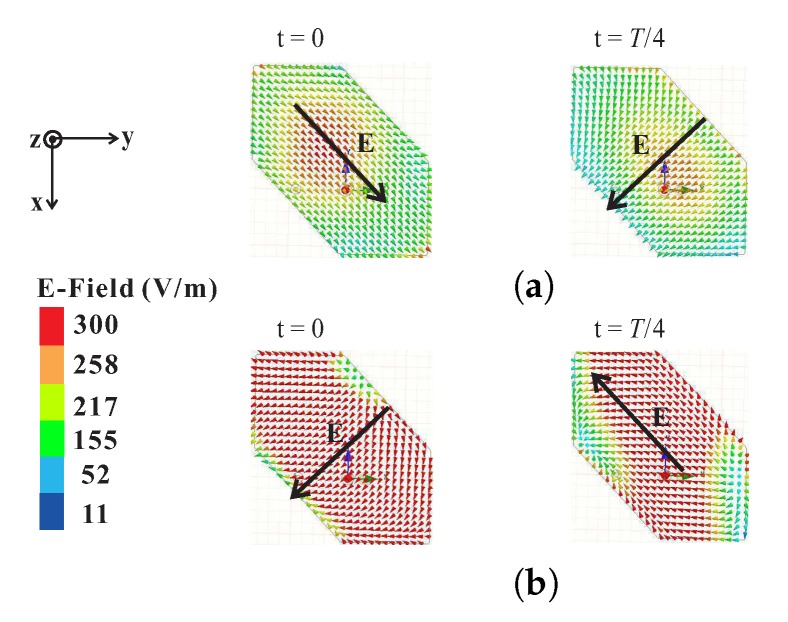
Simulated electric field distributions at the top of a hexagonal DR with time period *T* at frequencies of (**a**) 2.32 GHz; and (**b**) 3.2 GHz.

To determine the sense of circular polarization, the electric field distributions with time period *T* were observed on the top surface of the hexagonal DR from the broadside direction (θ = 0) at 2.32 GHz and 3.2 GHz, as depicted in Figure 5. In Figure 5a, the electric field vector *E* for t = 0 is pointing towards the lower right corner of the hexagonal DR. For t = *T*/4, at the same frequency, the electric field *E* rotates by 90 in the clockwise direction and is aligned with the center of the lower left truncated corner of the hexagonal DR. Similarly, in Figure 5b, the vectors *E* for t = 0 and t = *T*/4 are orthogonal to each other with a clockwise sense of rotation. Therefore, the proposed antenna exhibits left-handed circular polarization (LHCP) radiation.

**Figure 6:**
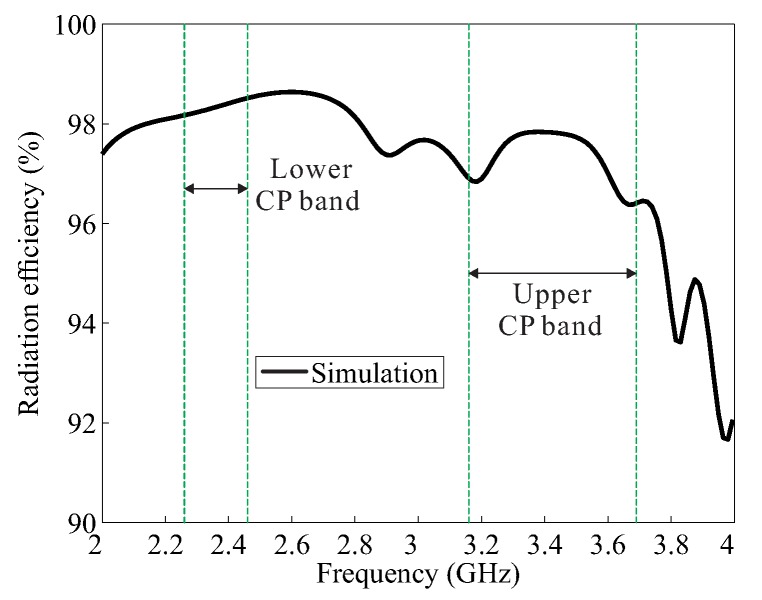
Simulated radiation efficiency of the proposed antenna.

Figure 6 presents a plot of the simulated radiation efficiency versus the frequency of the proposed antenna for the broadside direction (θ = 0), indicating that the simulated values are greater than 96% within the CP bands.

## 3. Measurement Results and Discussion

**Figure 7:**
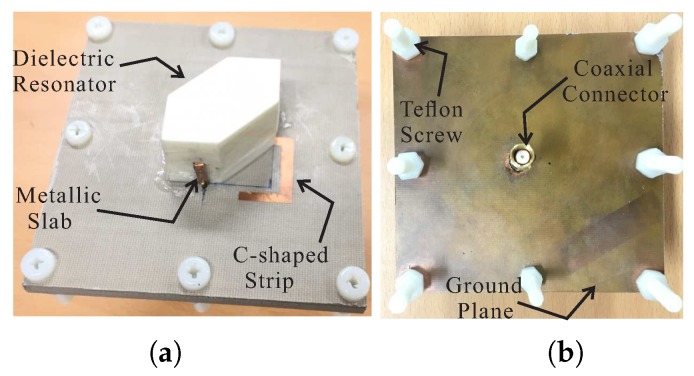
Photograph of the proposed antenna: (**a**) panoramic view and (**b**) back view.

**Figure 8:**
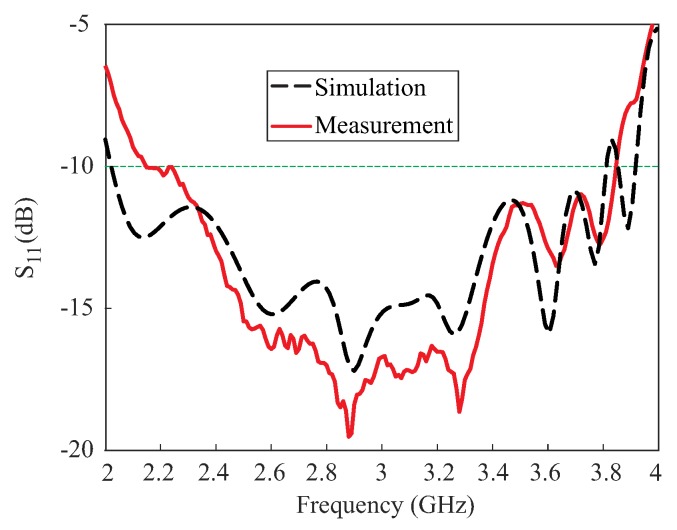
Measured and simulated reflection coefficients of the proposed antenna.

**Figure 9:**
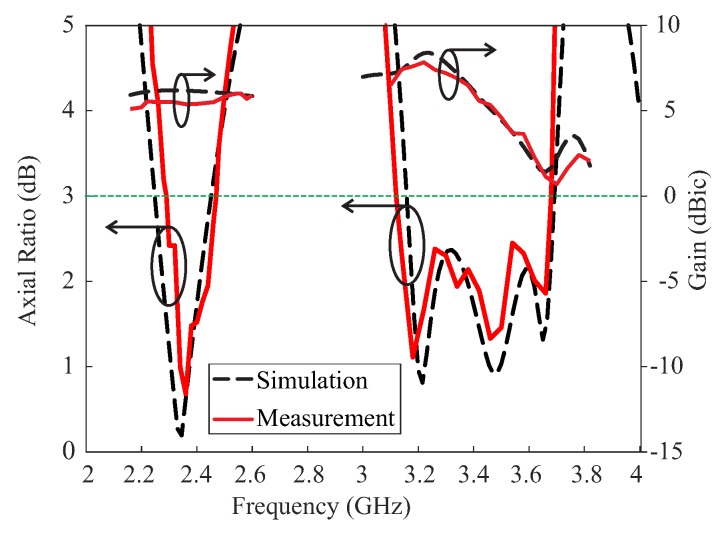
Measured and simulated axial ratios and left-handed circular polarization (LHCP) gains of the proposed antenna.

Based on the values in Table 1, a prototype of the proposed antenna was fabricated for experimental verification. Figure 7 presents a photograph of the proposed antenna. The reflection coefficients were measured by an Agilent 8510C network analyzer. As shown in Figure 8, the proposed antenna attained measured and simulated −10 dB reflection bandwidths of 56.43% (2.15–3.84 GHz) and 61.2% (2.03–3.82 GHz), respectively. The mismatch between the measured and simulated results at the lower frequencies can be attributed to fabrication imperfections. The AR, LHCP gain, and radiation patterns were evaluated in an RF anechoic chamber where a dual-polarized horn antenna was utilized. Far-field measurements were conducted for the frequency range of (2.1–3.8 GHz) with a step size of 20 MHz. A standard input power of 10dBm was applied and an RF power calibration is conducted using standard horn antennas. Figure 9 shows the results of the comparison between the measured and simulated ARs and LHCP gains of the proposed antenna for the broadside direction (θ = 0). The proposed antenna achieved measured 3 dB ARBWs of 7.56% (2.29–2.47 GHz) and 16.47% (3.12–3.68 GHz) compared to the simulated values of 8.47% (2.26–2.46 GHz) and 15.47% (3.16–3.69 GHz) in the lower and upper bands, respectively. Measured peak gains of 5.6 dBic and 7.84 dBic were obtained at frequencies of 2.32 GHz and 3.22 GHz, respectively, within the CP bands. It was also noted that the measured 3 dB ARBWs were fully covered within the measured −10 dB reflection bandwidth and are available for CP applications.

**Figure 10:**
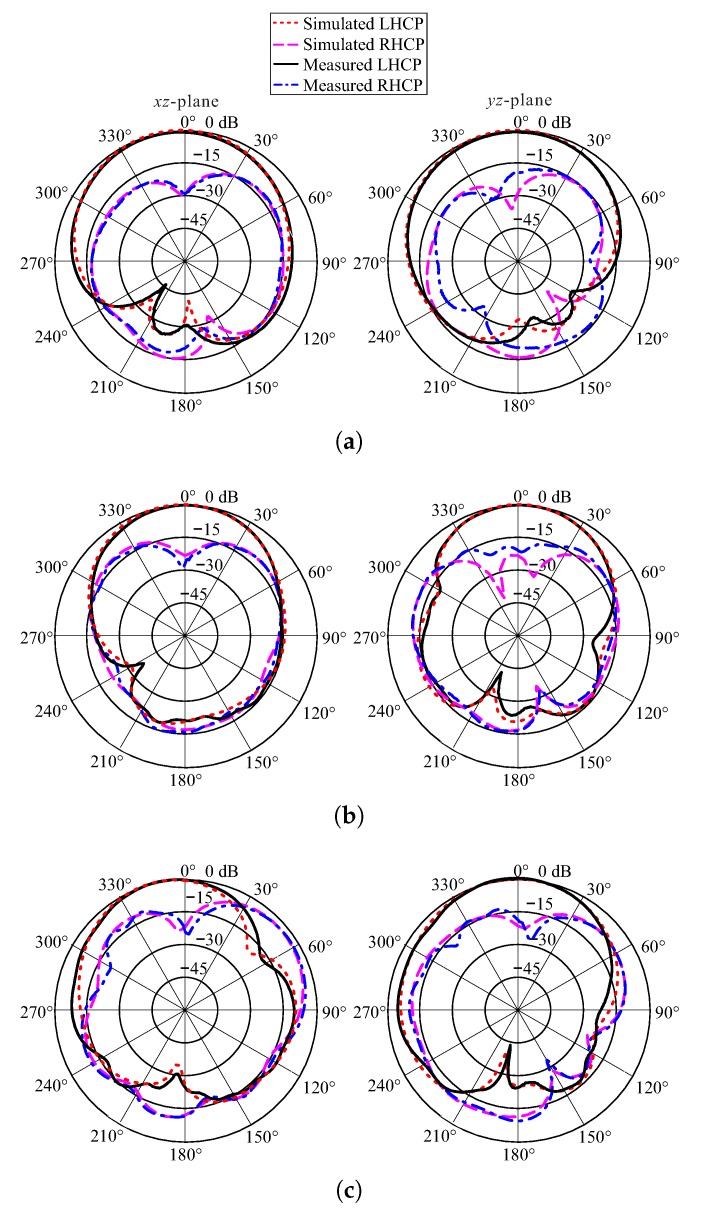
Measured and simulated normalized radiation patterns of the proposed antenna at (**a**) 2.36 GHz; (**b**) 3.18 GHz; and (**c**) 3.46 GHz.

The measured ARs minima lay at the frequencies of 2.36 GHz, 3.18 GHz, and 3.46 GHz. At these frequencies, the measured and simulated normalized radiation patterns of the proposed antenna on two cutting planes, the xz-plane and the yz-plane, are depicted in Figure 10. On both cutting planes for three frequencies, the LHCP gain was higher than the right-handed circular polarization (RHCP) gain by more than 19 dB in the broadside direction.

Finally, a comparison was made between the proposed antenna and those in earlier published works [13,14,15,16,17], as shown in Table 3. Compared with two studies [13,14], the proposed antenna possessed wider 3 dB ARBWs on both CP bands along with higher gains and a more compact height of 0.22$\lambda_{0}$ compared to the second study [14]. Note that the height includes the heights of the DR and the substrate of each design and that $\lambda_{0}$ represents the wavelength at the center frequency of the lower CP band. The proposed work offers superior performance compared to other outcomes [15,16,17] in terms of the upper 3 dB ARBW and peak gains on both CP bands, as well as a more compact height compared to the latter study [17]. In short, the proposed work offers overall good performance in terms of the 3 dB ARBWs, peak gains, and height compared to the aforementioned works.

## 4. Conclusions

A dual-band CP DRA designed on multi-layer substrates was presented. It was confirmed through the DWM that three pairs of orthogonal TE-modes, consisting of TE${}_{111}^{x}$, TE${}_{111}^{y}$, TE${}_{113}^{x}$-like, TE${}_{113}^{y}$-like, TE${}_{121}^{x}$-like and TE${}_{211}^{y}$-like modes, are separately excited in the hexagonal DR by the proposed 3-D meandered probe feeding. By introducing an asymmetric C-shaped metallic strip in the proposed design, a new CP band was induced which combined with the pairs of higher broadside and even TE-modes to yield a wide 3 dB ARBW at the upper band. A prototype of the proposed design was fabricated for practical evaluation. The proposed design attained a measured −10 dB reflection bandwidth of 56.43% (2.15–3.84 GHz) along with dual-band circular polarization with 3 dB ARBWs of 7.56% (2.29–2.47 GHz) and 16.47% (3.12–3.68 GHz). Radiation patterns were noted at the measured minima of the ARs. It was found that on both cutting planes, the LHCP gain was higher from RHCP by more than 19 dB in the broadside direction. In short, the proposed antenna, given its reasonably wide 3 dB ARBWs and high gains on both CP bands along with the absence of backward radiation, is a potential candidate for applications such as digital audio radio service (DARS) broadcasting (2.31–2.36 GHz), bluetooth/WLAN (2.4–2.484 GHz), and WiMAX (3.2–3.8 GHz). 

## Figures and Tables

**Table 1:** Geometric parameters of the proposed antenna.

Parameters	Values (mm)	Parameters	Values (mm)
a	31.4	$L_{1}$	12.1
b	33.1	$L_{2}$	3.1
d	25	$L_{3}$	17.7
g	3.60	$L_{4}$	10.2
h	1.52	$L_{5}$	3.5
$m_{1}$	5.22	$L_{6}$	11.5
w	3	$L_{7}$	24
$d_{1}$	12	$w_{1}$	1.7
$d_{v}$	1.2	$w_{2}$	1
$d_{x}$	5.78	$w_{3}$	2.4
$d_{y}$	2.3	$w_{4}$	3.2
$g_{x}$	100	$w_{5}$	3
$g_{y}$	104		

**Table 2:** Calculated dielectric waveguide model (DWM) and simulated High-Frequency Structure Simulator (HFSS) resonance frequencies of different TE-modes excited in the hexagonal dielectric resonator (DR).

$\varepsilon_{r}$	TE-Modes	f${}_{\mathrm{calc}.}$ (GHz)	f${}_{c}$ (GHz)	f${}_{\mathrm{simu}.}$ (GHz)
$\varepsilon_{eff}$	TE${}_{111}^{x}$	2.24	2.265	2.32
	TE${}_{111}^{y}$	2.29		
	TE${}_{113}^{x}$	3.85	3.86	3.2
	TE${}_{113}^{y}$	3.87		
	TE${}_{121}^{x}$	3.66	3.73	3.77
	TE${}_{211}^{y}$	3.8		
$\varepsilon_{d}$	TE${}_{113}^{x}$	3.39	3.395	3.2
	TE${}_{113}^{y}$	3.40		

**Table 3:** Comparison of the proposed antenna with those used in earlier published works.

Structure	Feeding Mechanism	Lower Band 3 dB ARBW (GHz)	Upper Band 3 dB ARBW (GHz)	Lower Band Peak Gain (dBic)	Upper Band Peak Gain (dBic)	Height ($\lambda_{0}$)
[13]	A rectangular slot-coupled DR	1.53–1.63 (6.3%)	2.40–2.49 (3.68%)	6.09	8.49	0.22
[14]	A cross slot-coupled DR	1.255–1.282 (2.1%)	1.538–1.572 (2.2%)	5.5	4.5	0.23
[15]	A rectangular slot-coupled DR with a pair of parasitic slots	4.75–5.5 (14.84%)	8.55–9.18 (7.11%)	4.3	5.8	0.19
[16]	A cross slot-coupled DR	3.08–3.6 (15.7%)	4.05–4.3 (6%)	2.3	4.7	0.13
[17]	A coaxial probe-fed DR	8.31–9.24 (10.6%)	10.18–11.66 (13.5%)	4.86	4.91	0.31
Proposed work	The 3-D meandered probe-fed DR	2.29–2.47 (7.56%)	3.12–3.68 (16.47%)	5.6	7.84	0.22
